# Chromium distribution in shoots of macrophyte *Callitriche cophocarpa* Sendtn.

**DOI:** 10.1007/s00425-014-2047-9

**Published:** 2014-03-05

**Authors:** Joanna Augustynowicz, Paweł Wróbel, Bartosz J. Płachno, Grzegorz Tylko, Zbigniew Gajewski, Dariusz Węgrzynek

**Affiliations:** 1 Unit of Botany and Plant Physiology, Faculty of Horticulture, Institute of Plant Biology and Biotechnology, University of Agriculture in Kraków, al. 29 Listopada 54, 31-425 Kraków, Poland; 2 Department of Medical Physics and Biophysics, Faculty of Physics and Applied Computer Science, AGH University of Science and Technology, al. Mickiewicza 30, 30-059 Kraków, Poland; 3 Department of Plant Cytology and Embryology, Faculty of Biology and Earth Sciences, Institute of Botany, Jagiellonian University, Gronostajowa 9, 30-387 Kraków, Poland; 4 Department of Cell Biology and Imaging, Faculty of Biology and Earth Sciences, Institute of Zoology, Jagiellonian University, Gronostajowa 9, 30-387 Kraków, Poland

**Keywords:** *Callitriche*, Chromium, EPXMA, Glands, Macrophytes, X-ray

## Abstract

The aim of the study was the analysis of Cr distribution in shoots of the macrophyte *Callitriche cophocarpa* by means of two X-ray-based techniques: micro X-ray fluorescence (μXRF) and electron probe X-ray microanalysis (EPXMA). Plants were treated with 100 μM (5.2 mg l^−1^) chromium solutions for 7 days. Cr was introduced independently at two speciations as Cr(III) and Cr(VI), known for their diverse physicochemical properties and different influence on living organisms. A comparative analysis of Cr(III)-treated plants by EPXMA and μXRF demonstrated high deposition of Cr in epidermal glands/hairs localized on leaves and stems of the plant shoots. Cr in Cr(III)-treated plants was recorded solely in glands/hairs, and the element was not present in any other structures. On the other hand, Cr in Cr(VI)-treated group of plants was rather found in vascular bundles. Moreover, the concentration of Cr in Cr(VI)-treated plants was significantly lower than in plants incubated in Cr(III) solution. The results obtained in this work suggest differences in chromium uptake, transport and accumulation dependent on the oxidative state of the element.

## Introduction

Excess of heavy metal ions in plant environment has a negative impact on the plant metabolism at different levels of plant functioning. In contrast to terrestrial plants, mechanisms related to the uptake or accumulation of heavy metal ions by aquatic ones are far less known. Due to the water environment of aquatic vascular plants (macrophytes), the availability of heavy metal compounds to their cells or tissues is much higher than in the case of terrestrial vegetation. Aquatic plants obtain minerals from both aquatic and sediment reservoirs. The uptake of metallic compounds by macrophytes depends on the chemical form of ions, and on the life form of particular plants: floating, emergent, submersed, well rooted or rootless (Malec et al. 2011). In the group of aquatic species, there are macrophytes that efficiently remove Cr contaminants, e.g.: *Eicchornia crassipes*, *Polygonum hydropiperoides*, *Nymphaea spontanea*, and *Leersia hexandra* (Choo et al. 2006; Qian et al. 1999; Zayed and Terry 2003; Zhang et al. 2007). Similarly to terrestrial plants, the Cr levels in shoots of aquatic species are in most cases lower than in roots, since root–shoot translocation of Cr is limited (Zayed and Terry 2003). For example, in the case of *Borreria scabiosoides* treated with Cr(III), the element is preferentially accumulated in cell walls and in some vacuoles of cortical parenchyma (Mangabeira et al. 2006). Moreover, species submersed in water may have a higher accumulating potential than floating or emergent ones due to the increased contact area with the surrounding environment (Rai et al. 1995). Thus, these species are of great interest for phytoremediation purposes.

In previous studies, we discovered the unusual ability of *Callitriche cophocarpa* Sendtn. to extract Cr from water solutions when the ions of the element were at Cr(VI) and Cr(III) oxidation states (Augustynowicz et al. 2010, 2013b). *Callitriche cophocarpa* belongs to the genus *Callitriche* (water starworts) that consists of about 50 globally distributed species, classified to the Callitrichaceae family. Species of *Callitriche* are aquatic, amphibious or terrestrial (Erbar and Leins 2004). The submersed *C. cophocarpa* is one of the most common *Callitriche* species in Europe (Schotsman 1972). Similarly to other species of the *Callitriche* genus, this plant is interesting due to its *geitonogamy*—a unique self-fertilization system (Philbrick and Bernardello 1992) and the potential utility of *C. cophocarpa* in phytoremediation of aquatic reservoirs (Favas et al. 2012; Pratas et al. 2010, 2012).

Cr(VI) and Cr(III) forms are the most stable and common in the environment. The main source of chromium relates to anthropogenic activity (e.g., metal and alloy manufacturing, brick lining, chrome plating, production of pigments and leather tanning), since the erosion of Cr-rich rocks is relatively low (Kabata-Pendias and Mukherjee 2007). Cr(VI) and Cr(III) differ in their physiochemical properties and, in consequence, in activities related to living organisms. Although Cr(III) at low concentrations is an essential microelement necessary for the glucose metabolism in mammals, its function in plants is not clear. Cr(VI), however, is a strong oxidizing agent toxic to biota (Saha et al. 2011; Zayed and Terry 2003). In aquatic systems, the levels of both speciations are often significantly over-limited (Kyzioł-Komosińska and Kukułka 2008) being harmful to aquatic life. Therefore, Cr(VI) and Cr(III) are treated by the Environmental Protection Agency (USA) as priority-toxic pollutants.

The concentration of Cr ions used in the present study induced some stress symptoms to *C. cophocarpa,* but did not cause serious physiological disorders (Augustynowicz et al. 2010, 2013b). Still, it was more than a hundred times higher than the concentration of Cr acceptable by Polish Ministry of the Environment regulations (Regulation, 9th of Nov 2011). The experimental conditions were environmentally relevant in the case of medium composition—filtered water from natural *C. cophocarpa* habitat and light intensity.

The objective of this work was to determine the distribution of chromium in leaves and stems of submersed macrophyte *C. cophocarpa* when Cr was administrated to plant environment as either Cr(VI) or Cr(III) forms. The distribution of the element was analyzed by means of two X-ray fluorescence-based methods. Micro X-ray fluorescence (μXRF) spectroscopy was used to determine the pattern of Cr accumulation in micrometer scale, whereas X-ray microanalysis combined with scanning electron microscopy (electron probe X-ray microanalysis; EPXMA) revealed submicrometer Cr distribution. Micro X-ray fluorescence is a non-destructive technique that allows studies of elements with a very narrow beam of X-rays at concentration levels in μg g^−1^ range (for review see Punshon et al. 2009). Electron probe X-ray microanalysis, however, enables elemental analysis with much higher level of detection (mg g^−1^), but gives the possibility of concomitant observation of specimen morphology (Goldstein et al. 2003). Until now, there were no available data dealing with chromium distribution in plants of the *Callitriche* genus. The obtained results will contribute to the knowledge concerning organ/tissue structures responsible for element uptake by aquatic phytoremediators.

## Materials and methods

### Plant material and incubation in Cr media

*Callitriche cophocarpa* was collected from the Dłubnia river, Southern Poland (50º16′N/19º56′E), during the vegetation season of 2012. Mature shoots about 10 cm long were rinsed with tap water several times followed by three times rinsing in distilled water. The Cr solutions were prepared using water derived from the natural environment of plants. River water was filtered (Supelco filters, 0.2 μm pore size) to prevent growth of microorganisms. The chemical composition of water was analyzed by means of inductively coupled plasma mass spectrometry (ICP-MS; ELAN 6100, Perkin Elmer, Waltham, MA, USA) (PN-EN ISO 9963-1:^2001^) and titration methods (PN-ISO 9297:^1994^, PN-EN ISO 17294-1:^2007^). The quantitative results were obtained with ICP multi-element standard (Merck). The concentrations of ions (mg l^−1^) present in water were the following: 4.24 Na^+^, 1.75 K^+^, 69.65 Ca^2+^, 5.01 Mg^2+^, 2·10^−3^ Fe^2+^, 5·10^−3^ Mn^2+^, 5·10^−3^ Zn^2+^, 6·10^−4^ Cu^2+^, 10^−3^ Mo^6+^, 16.50 Cl^−^, 10.20 SO_4_^2−^, 189.00 HCO_3_^2−^, 13.50 NO_3_^2−^, 0.15 PO_4_^3−^, 0.08 BO_3_^3−^. The level of Pb, Hg, and Cd did not exceed 0.2 μg l^−1^ and Cr content was lower than 0.02 μg l^−1^. The electrical conductivity of water was equal to 0.335 mS cm^−1^, pH 7.8 and Eh = 180 mV. The solutions containing 100 μM (5.2 mg l^−1^) of Cr(VI) or Cr(III) were prepared from K_2_Cr_2_O_7_ and Cr_2_(SO_4_)_3_18H_2_O, respectively (POCh Gliwice, Poland). 1.5 g of shoots were cultured in 300 ml of the aforementioned Cr solutions or in the control solution (without Cr salts) for 7 days in the phytotron under the 16 h of light intensity at 35 μmol m^−2 ^s^−1^ (LF 36 W/54, Piła, Poland) and 8 h of darkness, at 23 °C. The light intensity was comparable to the one detected in the natural *Callitriche* environment. The analysis of chromium distribution was performed on mature leaves and stems.

### μXRF of chromium

The plant samples were prepared according to a freeze-drying protocol to avoid dehydration and redistribution of Cr ions during prolonged μXRF measurements. After treatment with Cr(VI)- and Cr(III)-containing media, the shoots were thoroughly washed in distilled water, gently dried with filter paper to remove access of water and immediately plunged-frozen in liquid nitrogen. Then, the samples were transferred to lyophilizer chamber (Alpha 1-4 Martin Christ Gefriertrocknungsan-lagen GmbH lyophilizer, Germany) and left for 24 h at 1.03 mbar and −20 °C. After drying, the temperature of the specimen holder was gradually increased to achieve room temperature and the plant samples removed. All specimens were finally mounted between two 2.5 μm mylar films stretched on the plastic holder and positioned on the motorized stage of μXRF machine.

Two-dimensional distribution maps of chromium or potassium (as a vascular bundle indicator) (Thompson and Zwieniecki 2005) were performed with a laboratory setup consisting of low-power X-ray tube (XOS, East Greenbush, USA) with molybdenum anode and SDD detector (Ketek, Munich, Germany) (Wróbel et al. 2012). The angle between the impinging beam and the sample normal was 45° and the angle between detector axis and the sample normal was 45°. The X-ray tube voltage and current were 50 kV and 1 mA, respectively. Primary radiation from the X-ray tube was focused with polycapillary lens into Gaussian-shaped beam (Węgrzynek et al. 2008). The size of the focal spot was 16.4 μm at full width of half maximum and the size of irradiated area was 380 μm^2^. The mapping of chromium was performed for the area of 1–1.5 mm^2^ with step size in *X*–*Y* direction equal to 20 μm and dwell-time 1–1.5 s. The average time of imaging of single sample was 4.5 h. Thus, the qualitative maps of Cr distribution obtained with μXRF present X-ray intensity (count per second) recorded by SDD detector from irradiated area. The intensity of X-ray emission is proportional to element content. Three (control) or eight (Cr-treated samples) independent leaves and stems were mapped.

### EPXMA of chromium

Leaves of *C. cophocarpa* were cut out from the stem, transferred to a drop of plant culture medium and divided into two parts perpendicularly to their long axis. Then, one group of leaf specimens was prepared for chromium analyses in their epidermal structures only—glands/hairs and stomata, whereas the second group was prepared for chromium investigation in mesophyll and vascular tissues. The specimens from the first group were gently dried with filter paper to remove excess of water and attached to aluminum specimen carriers (no. 16701950, Leica Microsystems, Germany) covered with a thin layer of tissue freezing medium (OCT Compound, Leica Microsystems, Germany). There, the specimens were positioned onto the carrier doubly to expose the upper and lower surface of the leaves. The specimens from the second analytical group were carefully enclosed in tissue freezing medium before fixation at low temperature. All samples were quickly plunged-frozen in solidified nitrogen (slash) at a temperature around −210 °C and stored in liquid nitrogen for further processing. The first group of frozen samples was transferred directly to the tissue dryer (Edwards ETD4, Edwards High Vacuum International, UK) in cold gas nitrogen atmosphere and lyophilized overnight at 0.01 mbar and −30 °C. After drying, the temperature of the specimen holder was gradually increased to achieve room temperature and the specimens removed.

The second group of samples was transferred to a cryostat chamber (CM1850 UV, Leica Microsystems, Germany), attached perpendicularly to the surface of cutting holders with freezing medium and trimmed until the internal structure of the leaves was exposed; normally 0.5 mm of the leaf was trimmed to visualize mesophyll and vascular tissues in scanning electron microscope (SEM). Then, the samples were transferred to the tissue dryer and lyophilized as mentioned above. Dried leaves were additionally attached to the aluminum specimen holders with current conductive carbon glue (SPI Supplies, USA), coated with a thin carbon layer (~15 nm) in a JEE 4B evaporator (JEOL, Tokyo, Japan), and analyzed in a JSM-5410 scanning electron microscope (JEOL) with a NORAN 679A- SES energy-dispersive spectrometer (EDS) equipped with a NORVAR thin-window (Noran Instruments, Middletown, WI, USA).

The EDS detector was positioned at take-off angle of 25° and 30 mm away from the beam interaction volume (solid angle 0.0333 sr). Preliminary qualitative measurements to determine minimum detection limit for Cr were performed at 15 keV accelerating voltage with the beam size of 80 nm and the probe current of 250 pA as measured by means of the Faraday cup. It made analyses possible with the count rate of 2,000 quanta per second for the deadtime value of ~20 %. Point analyses of mesophyll and vascular tissues were performed to ascertain the presence of chromium. Mapping of chromium distribution was performed for leaf regions with glands/hairs and stomata at the same geometry of the analytical system. However, to obtain sufficient intensity of characteristic X-rays for chromium, the beam size of 130 nm was used. Thus, the probe current increased to 800 pA and 4,500 counts per second were registered by EDS detector. Maps of Cr distribution were created for all experimental groups when 100 frames were accumulated by the system at the resolution of 512 × 512 pixels. Three samples of each experimental group were mapped.

### Plant morphology examination—light microscopy

Fresh as well as ethanol-fixed (70 % ethanol solution) plant material was hand-sectioned with a razor-blade and examined under an Olympus BX60 (Olympus Corporation, USA) microscope equipped with differential interference contrast (DIC). Image-Pro PLUS ver.4.0 (Media Cybernetics Inc., Rockville, MD, USA) software was applied to measure distances between epidermal glands/hairs in microscopic images. At least six independent leaves/stems were used for analysis.

### Statistics

Three independent sets of experiments were conducted, with each set comprising several independent replicates. Results were statistically verified based on STATISTICA 10 software. The statistical tests were chosen according to the distribution of results. Non-parametric Kruskal–Wallis/Mann–Whitney *U* tests were applied to compare differences between objects. Following the rejection of null hypothesis, non-parametric multiple comparison test (Dunn’s test) was performed to determine statistical significance of results at *α* = 0.05.

## Results

**Fig. 1:**
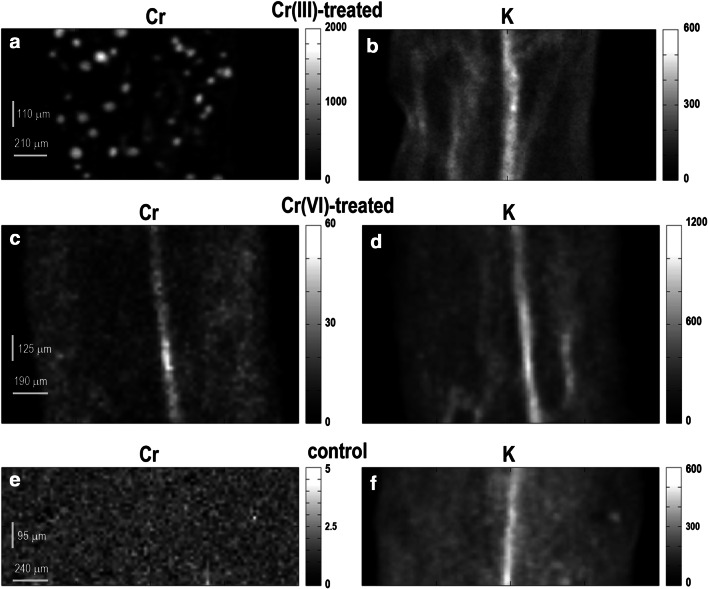
Representative μXRF maps of chromium and potassium distribution in leaves of Cr(III)- and Cr(VI)-treated as well as control group of *C. cophocarpa*. **a** Cr(III)-treated leaf shows spot-like structures with high concentration of Cr; **b** *K* distribution in Cr(III)-treated leaf that visualizes vascular bundle region; **c** Cr(VI)-treated leaf with Cr accumulated in the region of vascular bundle; **d** *K* distribution in Cr(VI)-treated leaf that visualizes vascular bundle region; **e** the level of background intensity of X-rays characteristic for chromium emission in a leaf from the control group of plants; **f** *K* distribution in a leaf of the control group of plant that visualizes vascular bundle region. *Grayscale* indicates intensity of X-ray signal (cps) characteristic for chromium emission energy

We found significant differences in Cr distribution in both groups of Cr-treated plants, i.e., in the plants exposed to Cr(III) and Cr(VI). Figure 1a and c shows representative maps of Cr accumulated in leaves of *C. cophocarpa*. The plants treated with Cr(III) ions revealed spot-like chromium distribution (Fig. 1a), whereas leaves obtained from plants treated with Cr(VI) (Fig. 1c) showed homogeneous accumulation of Cr with significantly higher Cr deposition in the region of vascular tissue as indicated by *K* pattern (Fig. 1b, d, f). The amount of Cr deposited in leaves in both Cr-treated plants differed significantly. The median X-ray intensity registered in Cr(III)-treated leaves was around 19-times higher in relation to leaves from Cr(VI)-treated plants (Table 1). There was no chromium registered in leaves obtained from the control group of plants (Fig. 1e).

**Table 1:** The median as well as minimal and maximal intensities of chromium characteristic X-rays emitted from leaves and stems not exposed to Cr (control) or exposed to Cr(III) or Cr(VI)

Specimen		X-ray intensity (counts per second)		
		Median	Min.	Max.
Cr(III)	Leaf	91.3 (d)	1.7	1,802.5
Cr(VI)		4.7 (b)	0.3	301.9
Control		1.1 (a)	0.2	4.7
Cr(III)	Stem	153.1 (e)	2.2	2,167.8
Cr(VI)		12.7 (c)	0.6	144.6
Control		1.2 (a)	0.2	3.3

The median value relates to X-ray intensity of fluorescence of Cr signal (counts per second) at the scanned area (380 μm^−2^). The letters indicate statistically significant differences between treatments (Kruskal–Wallis non-parametric ANOVA and Dunn’s test; *α* = 0.05)

**Fig. 2:**
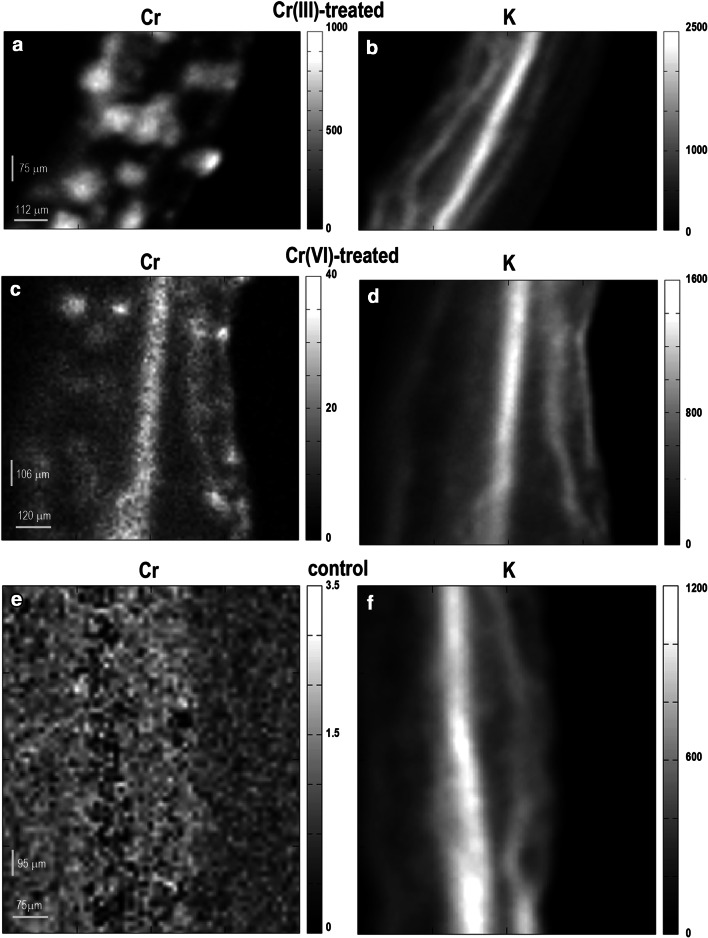
Representative μXRF maps of chromium and potassium distribution in stems of Cr(III)- and Cr(VI)-treated as well as control group of *C. cophocarpa*. **a** Cr(III)-treated stem shows spot-like structures with high concentration of Cr; **b** *K* distribution in Cr(III)-treated stem that visualizes vascular bundle region; **c** Cr(VI)-treated stem with Cr accumulated in the region of vascular bundle; **d** *K* distribution in Cr(VI)-treated stem that visualizes vascular bundle region; **e** the level of background intensity of X-rays characteristic for chromium emission in a stem from the control group of plants; **f** *K* distribution in a stem of the control group of plant that visualizes vascular bundle region. *Grayscales* indicate intensity of X-ray signal (cps) characteristic for chromium emission energy

Similarly to the leaves, mapping of stems from both groups of Cr(III)-treated plants showed the presence of spot-like structures (Fig. 2a). The maps of Cr(VI)-treated stems revealed Cr-rich areas (Fig. 2c) in the region of vascular bundle as indicated by *K* (Fig. 2b, d, f). Similarly to the leaves, the median X-ray intensity of Cr signal in stems subjected to Cr(III) was 12-times fold higher than in stems treated with Cr(VI). There was no chromium registered in stems obtained from the control group of plants (Fig. 2e).

**Fig. 3:**
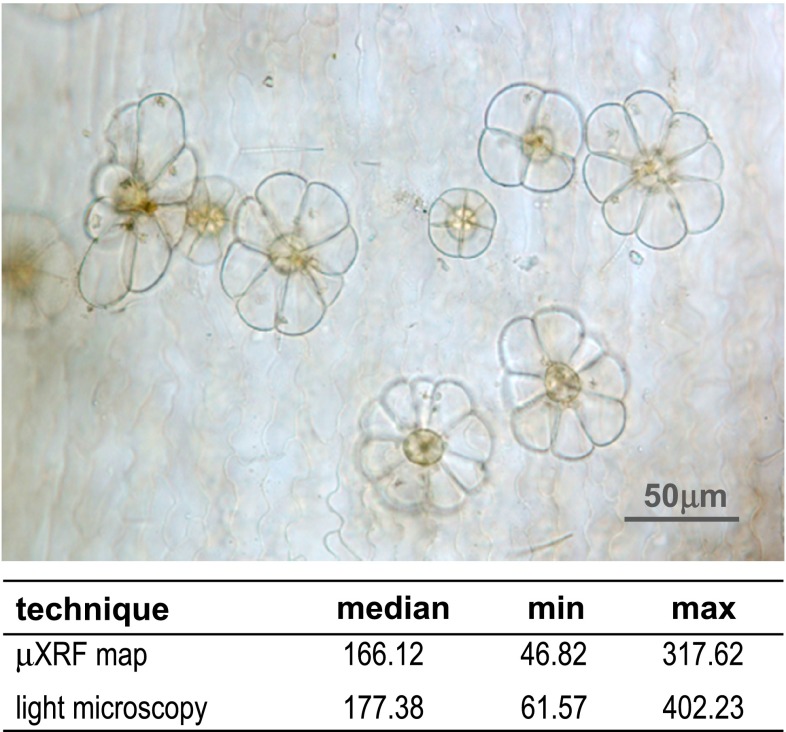
Light microscopy photograph of *C. cophocarpa* leaf epidermis with well visible glands/hairs. The table represents median as well as minimal and maximal distances (in μm) between epidermal glands/hairs measured on the basis of light micrographs and those measured between spot-like structures registered in μXRF maps. No statistical differences between the values were found (Mann–Whitney *U* test; *α* = 0.05)

In the next step of the study, distances between spot-like structures in leaves subjected to Cr(III) were measured on the basis of the μXRF maps and compared to distances obtained from light microscopic photographs of leaf surfaces (Fig. 3). The measurements were carried out only on flat surfaces of leaves because the geometry of stems (round in shape) made the measurements inaccurate. Many epidermal multi-cellular hairs were observed on both lower and upper leaf epidermis (Fig. 3). The median distance between investigated structures observed in light microscope and in μXRF maps was not statistically different. It suggested that Cr-contained spot-like structures were probably glands/hairs present on the upper or lower epidermis.

**Fig. 4:**
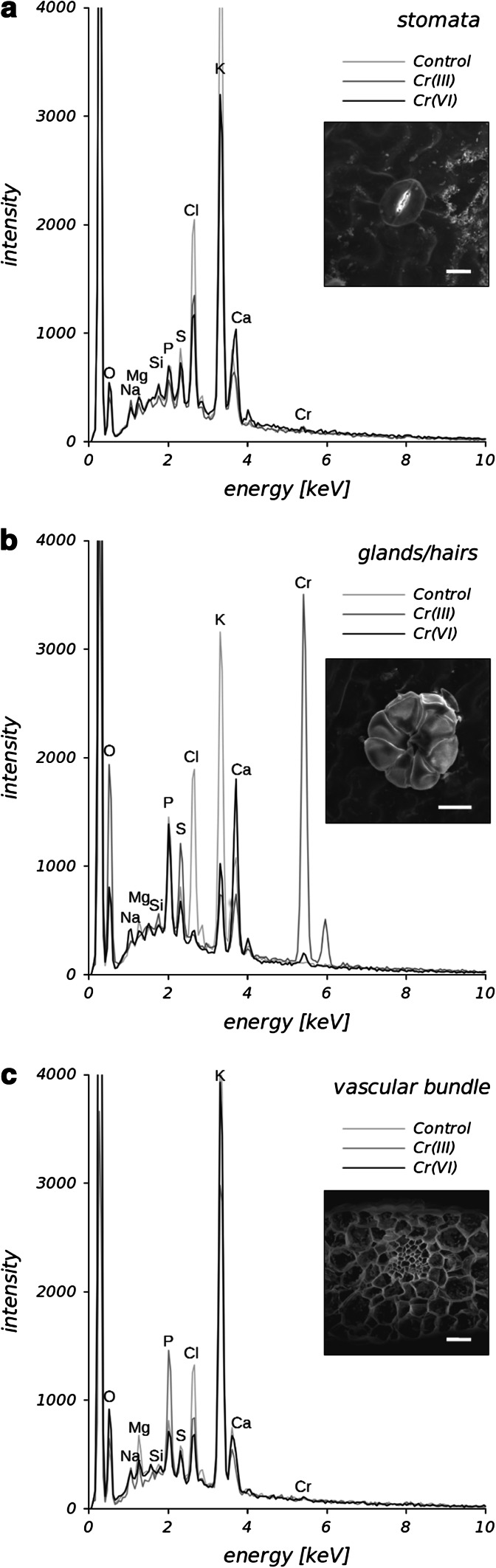
Representative spectra obtained after EPXMA of stomata cells (**a**), glands/hairs (**b**) and vascular bundle cell walls (**c**) using point mode analysis with concomitant imaging of structures of interest (inserts). The elements present in spectra are marked with their symbols. Analysis revealed chromium accumulation above the limit of detection (<0.2 mass%) only in glands/hairs of leaf or stem samples (**b**) but below this limit in stomata (**a**) and vascular bundle cells (**c**). Note the evident emission of chromium when analysis concerned glands/hairs derived from plants treated with Cr(III) solution and significantly lower intensity of chromium X-rays in the case of these structures from Cr(VI)-treated plants. *Bar *30 µm

**Fig. 5:**
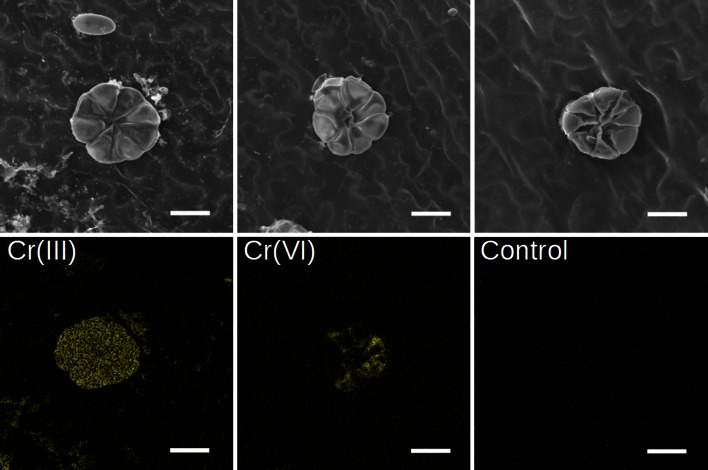
Representative qualitative maps of chromium distribution in glands/hairs of Cr-treated and untreated (control) plants. Please note that the intensity of X-rays is not scaled for the presented images, thus the maps cannot be compared to reveal differences in chromium content between the experimental groups. *Bar* 30 μm

To confirm that glands/hairs are responsible for Cr accumulation in Cr(III)-treated plants, SEM equipped with X-ray energy-dispersive detector was applied. Concomitant SEM examination and X-ray analysis of Cr in *C. cophocarpa* stomata or glands/hairs confirmed the concentration of chromium below the limit of detection for EPXMA analysis (<0.2 mass%) in the stomata cells (Fig. 4a) and its presence in glands/hairs when the plants were treated with chromium (Fig. 4b). It is noteworthy that the intensity of Cr X-rays emitted from glands/hairs of Cr(III)-treated plants was significantly higher than the intensity detected for these structures of Cr(VI)-treated plants (Fig. 4b). Analysis of Cr in the vascular bundles of leaves or stems by means of EPXMA did not show X-ray emission at the energy of Cr-*Kα* line (Fig. 4c; chromium concentration below the detection limit). Further SEM examination combined with X-ray mapping of chromium revealed that indeed the element is accumulated in epidermal glands/hairs of leaves and stems from both Cr-treated groups of plants (Fig. 5). Cr was distributed homogeneously in epidermal glands/hairs of Cr(III)-treated plants, but its accumulation in these structures of Cr(VI)-treated group was heterogeneous. No chromium was detected in glands/hairs of the control group of plants.

## Discussion

The methods used in this study enabled to determine Cr accumulation patterns in shoots of *C. cophocarpa* treated with two different chromium ions—at third and sixth oxidative states. A comparative analysis of Cr(III)-treated plants by EPXMA and μXRF demonstrated high deposition of Cr in epidermal glands/hairs localized on leaves and stems of the plant shoots. Cr in Cr(III)-treated plants was recorded solely in glands/hairs, and the element was not present in any other structures. On the other hand, Cr in Cr(VI)-treated group of plants was rather found in vascular bundles. This phenomenon could be explained on the basis of physicochemical properties of Cr. The ionic form of Cr in water solution depends on several factors, e.g., pH, ionic strength, presence of complexing agents (organic matter) and redox potential (Eh). In the absence of complexing agents and pH range of 4–10, the dominant forms of Cr(III) are cations, e.g., CrOH^2+^ or Cr_2_(OH) _2_^4+^. Cr(III) in the cationic form passively diffuses across the cell wall and plasma membranes of cells, easily binding to hydroxyl, carboxyl, amide or sulfhydryl groups (Zayed and Terry 2003). Cr(III) exhibits strong affinity to organic ligands, like phenolic derivatives, what leads to formation of chromium-derived complexes (Kotaś and Stasicka 2000; Kyziol et al. 2006).

In general, many aquatic and amphibious plants have epidermal secretory hairs (trichomes/glands) on their shoots, e.g., *Nymphaea* (Lavid et al. 2001a; Lavid et al. 2001b), *Myriophyllum* (Godmaire and Nalewajko 1990), *Aldrovanda* (Lloyd 1942), *Utricularia* (Płachno and Świątek 2010), and also *Callitriche* (Erbar and Leins 2004). The hairs secrete substances into the external environment, but for example in *Nymphaea*, they may also be responsible for ion absorption from the surrounding water (Lavid et al. 2001a; Lüttge and Krapf 1969). It was also shown that in some aquatic plants the hairs immobilize toxic heavy metals (Lavid et al. 2001a, b). It seems that glands/hairs of *C.* *cophocarpa* have a similar function. The study shows for the first time the possible role of epidermal hairs in Cr(III) accumulation in the leaves and stems of *C. cophocarpa*. Moreover, in the previous work by Augustynowicz et al. (2013b), it was revealed that the major pool of Cr(III) ions follows the strongest mechanism of metal binding to the organic matter in shoots of *C. cophocarpa.* Cr(III) bound in the form of chelates/complexes with surface organic groups. Thus, we postulate that Cr(III) is strongly accumulated only in the epidermal glands/hairs and these structures act as regulators of heavy metal ion exchange between plant tissues and water environment. In our opinion, the glands/hairs might block the transfer of Cr(III) ions to internal parts of shoots playing the role of natural barrier for chromium ions or other high atomic number elements (Lavid et al. 2001a, b).

In aquatic solutions, Cr(VI) exists as anion, mainly dichromate (Cr_2_O_7_^2−^) or chromate (CrO_4_^2−^). Cr(VI) is very mobile in the broad range of pH, thus diverse mechanisms of Cr(VI)/Cr(III) transfer and accumulation in plants must be engaged. Cr(III) uptake is a passive process whereas Cr(VI) is transported under the control of sulfate (anionic) transporters (Appenroth et al. 2008; Kaszycki et al. 2005; Kotaś and Stasicka 2000). It seems that the easily mobile Cr(VI) ions are transported in the plant via the vascular bundle, since we found chromium in this tissue when *C. cophocarpa* was treated with Cr(VI) ions. Cr(VI) is weakly bound to the organic matter of this plant. 34 % of Cr(VI) ions was found in water soluble fraction, whereas 23 % in mobile (easily exchangeable) fraction (Augustynowicz et al. 2013b). Since the sorption capacity of Cr(VI) is far less pronounced than of Cr(III), the anions can stay dissolved. Recently, Xue et al. (2010) found xylem- and phloem-based translocations of copper in *Hydrilla verticillata*. Similarly, intensive cadmium transport has been also observed in four emergent wetland species (Zhang et al. 2010). Still, detailed mechanisms of transport of heavy metal ions in the vascular system of macrophytes remain unknown (Malec et al. 2011).

Some amounts of Cr were also detected in glands/hairs of leaves and shoots of *Callitriche* when subjected to Cr(VI). Still, the level of Cr detected in leaves and stems of Cr(VI)-treated *C. cophocarpa* was significantly lower than in Cr(III)-treated plants. These results are consistent with the previous data obtained by means of inductively coupled plasma optical emission spectrometry (ICP-OES) (Augustynowicz et al. 2013b). However, the reported technique registers element concentrations only, without giving an insight into its distribution in tissues or cells. Lavid et al. (2001b) studied *Nymphea* exposed to Cr(VI) salts. They revealed that epidermal glands exhibited high Cr contents, which correlated with the increased amount of polyphenols: hydrolyzable tannins, gallic and tannic acids. It is probably the same strategy of chromium binding in *C. cophocarpa* glands/hairs. Recently, high concentrations of cinnamic acid-derivates as well as flavonoids were found in *C. cophocarpa* (Augustynowicz et al. 2014). It must be pointed out that chromium at the sixth oxidative state Cr(VI) could be reduced to the third oxidative state Cr(III), which is also widely considered as the main bioremediation strategy of Cr(VI) (Zayed and Terry 2003). Augustynowicz et al. (2013a) found internal Cr(VI) reduction in shoots of *C. cophocarpa* by means of the electron paramagnetic resonance spectroscopy (*L*-band EPR). Reduction of Cr(VI) to Cr(III) may be a reason of some Cr accumulation in epidermal glands of Cr(VI)-treated *Nymphea* plants (Lavid et al. 2001b) as well as in individual glands/hairs of *Callitriche* stems and leaves.

Concluding, our study has shown different distribution patterns of Cr in *C. cophocarpa* shoots related to their oxidation state. Cr is exclusively accumulated in epidermal glands/hairs when Cr(III) is present in plant environment. However, in the case of Cr(VI) exposure, the element is principally found in the vascular bundles.
